# Design of the exhale airway stents for emphysema (EASE) trial: an endoscopic procedure for reducing hyperinflation

**DOI:** 10.1186/1471-2466-11-1

**Published:** 2011-01-07

**Authors:** Pallav L Shah, Dirk-Jan Slebos, Paulo FG Cardoso, Edward J Cetti, Gerhard W Sybrecht, Joel D Cooper

**Affiliations:** 1 Royal Brompton Hospital, Sydney Street, London SW3 6NP, UK, and Imperial College London, UK; 2 University Medical Center Groningen, 9700 RB Groningen, The Netherlands; 3 Heart Institute (InCor)-Hospital das Clínicas da Faculdade de Medicina da Universidade de São Paulo, Rua Dr.Eneas de Carvalho Aguiar 44, 2 andar, bloco 2, sala 9, São Paulo-SP, Brazil; 4 Klinik fur Innere Medizin, Pneumologie, Allergologie, Beatmungs- und Umweltmedizin, Universitätsklinikum des Saarlandes, Germany; 5 Hospital of the University of Pennsylvania, 3400 Spruce St, Philadelphia, PA, 19104, USA

## Abstract

**Background:**

Airway Bypass is a catheter-based, bronchoscopic procedure in which new passageways are created that bypass the collapsed airways, enabling trapped air to exit the lungs. The Exhale Airway Stents for Emphysema (EASE) Trial was designed to investigate whether Exhale^® ^Drug-Eluting Stents, placed in new passageways in the lungs, can improve pulmonary function and reduce breathlessness in severely hyperinflated, homogeneous emphysema patients (NCT00391612).

**Methods/Design:**

The multi-center, randomized, double-blind, sham-controlled trial design was posted on http://www.clinicaltrials.gov in October 2006. Because Bayesian statistics are used for the analysis, the proposed enrollment ranged from 225 up to 450 subjects at up to 45 institutions. Inclusion criteria are: high resolution CT scan with evidence of homogeneous emphysema, post-bronchodilator pulmonary function tests showing: a ratio of FEV_1_/FVC < 70%, FEV_1_≤50% of predicted or FEV_1 _< 1 liter, RV/TLC≥0.65 at screening, marked dyspnea score ≥2 on the modified Medical Research Council scale of 0-4, a smoking history of at least 20 pack years and stopped smoking for at least 8 weeks prior to enrollment. Following 16 to 20 supervised pulmonary rehabilitation sessions, subjects were randomized 2:1 to receive either a treatment (Exhale^® ^Drug-Eluting Stent) or a sham bronchoscopy. A responder analysis will evaluate the co-primary endpoints of an FVC improvement ≥12% of the patient baseline value and modified Medical Research Council dyspnea scale improvement (reduction) ≥1 point at the 6-month follow-up visit.

**Discussion:**

If through the EASE Trial, Airway Bypass is shown to improve pulmonary function and reduce dyspnea while demonstrating an acceptable safety profile, then homogeneous patients will have a minimally invasive treatment option with meaningful clinical benefit.

**Trial Registration:**

ClinicalTrials.gov: NCT00391612

## Background

The primary objective of the EASE randomized, double-blind study is to evaluate the safety and effectiveness of the Exhale^® ^Drug-Eluting Stent (Broncus Technologies, Mountain View, CA) in homogeneous emphysema subjects with severe hyperinflation.

According to the National Center for Health Statistics, over 3.7 million adults in the United States report being diagnosed with emphysema, a chronic, progressive, irreversible disease of the lungs (1). It is an under-diagnosed and incurable disease often associated with chronic bronchitis and conditions such as pulmonary hypertension and heart failure. Emphysema patients suffer from hyperinflation because of the decrease in the elastic recoil of the lungs which, along with airway collapse, increases expiratory flow resistance. This is reflected in an increase in residual volume (RV), a reduction in expiratory flows (as measured by forced vital capacity [FVC] and forced expiratory volume in 1 second [FEV_1_]) and an increase in dyspnea. In end-stage emphysema, even a mild exacerbation can cause the patient's condition to deteriorate rapidly with profound hypoxemia, hypercapnia, and respiratory acidosis.

There is currently no cure for emphysema and the goal of treatment is primarily to relieve symptoms and reduce exacerbations. For patients with a heterogeneous pattern of emphysema and upper lobe predominance, lung volume reduction surgery can offer significant benefit but with morbidity and mortality. Lung transplantation is a widely-accepted surgical treatment for homogeneous (diffuse) emphysema. It is, however, an unrealistic option for most patients as lung transplant eligibility is limited both by stringent patient selection criteria and the scarcity of donor lungs. In the US in 2009, approximately 434 lung transplants were performed because of emphysema/COPD (2). A minimally invasive treatment that improves pulmonary function and reduces dyspnea in patients with homogeneous emphysema would provide a significant new option for these patients. Airway Bypass is a bronchoscopic procedure currently under evaluation to determine if creating small extra-anatomic openings between the diseased lung and the distal bronchi can reduce hyperinflation in homogeneous emphysema patients.

The EASE Trial is a multi-center, randomized, double-blind, sham-controlled study design. The endpoints for safety and effectiveness are measured at 6 months after the procedure.

## Methods/Design

The ethics committee of the participating centers approved the trial and all subjects signed an informed consent prior to entering the study. A minimum of 225 and a maximum of 450 subjects can be randomized under this Bayesian study design. All participants receive standard medical management for the duration of the study. Each subject who meets all eligibility criteria is randomized to one of the two groups, receiving the Exhale Drug-Eluting Stents or sham bronchoscopy, assigned 2:1 using a computer program.

**Figure 1:**
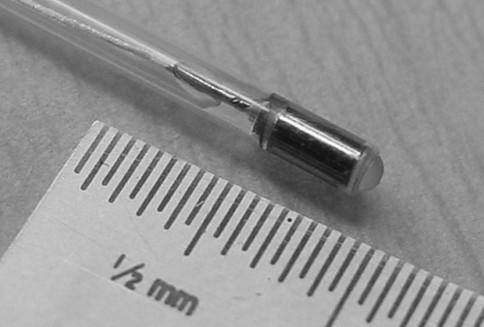
**Exhale Doppler probe tip**.

The Exhale Drug-Eluting Stent (DES) is used in the Airway Bypass procedure with the Exhale Doppler System, the Exhale^® ^Transbronchial Dilation Needle and commercially available inflation syringes and bronchus-blocking balloons. All of the catheters fit in a bronchoscope working channel of 2 mm or larger. The Exhale^® ^Doppler Probe (Figure 1), when connected to the Doppler Processing Unit (DPU) enables the physician to eliminate potential Airway Bypass sites which are associated with an adjacent blood vessel. The long flexible Doppler catheter is inserted through the working channel of a bronchoscope and has a 1.4 mm diameter, 8 MHz ultrasound transducer at the distal tip. At its proximal end a connector plugs into the DPU.

**Figure 2:**
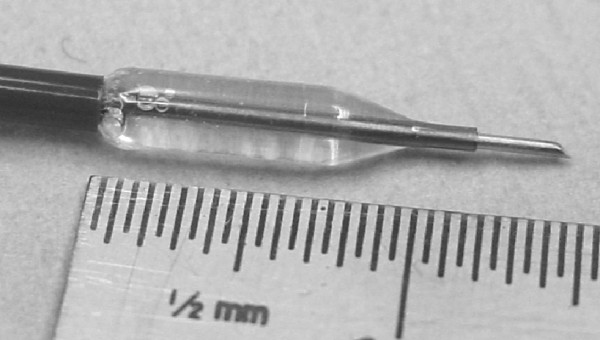
**Exhale Transbronchial Dilation Needle (extended tip), with the dilation balloon inflated**.

The transbronchial dilation needle (Figure 2) pierces the airway wall and dilates the passage, allowing placement of the Exhale DES. The device is comprised of a dilation balloon and a 25 gauge needle that extends up to 4 mm beyond the distal end of the balloon. Following passage creation, the area around the passage is rescanned by using the Doppler probe to further reduce the risk of encountering a blood vessel during stent placement.

**Figure 3:**
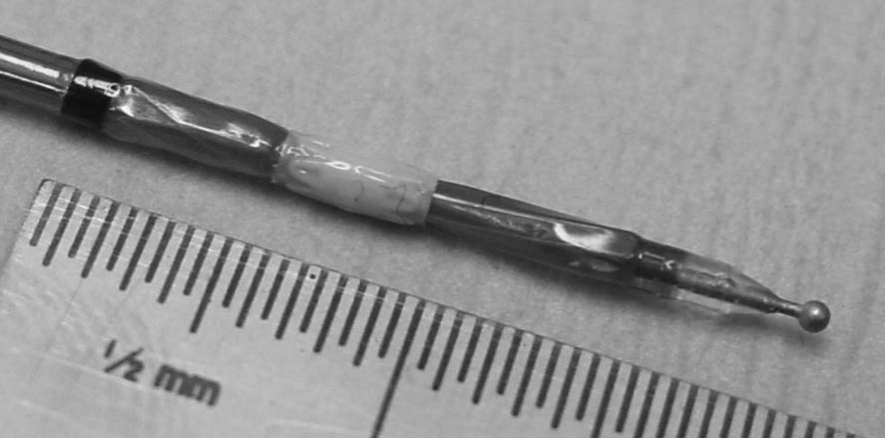
**Exhale Drug-Eluting Stent (white), and underlying balloon mounted on the delivery catheter**. The rounded tip at the end of the catheter makes it easier to place the stent catheter in the passage.

**Figure 4:**
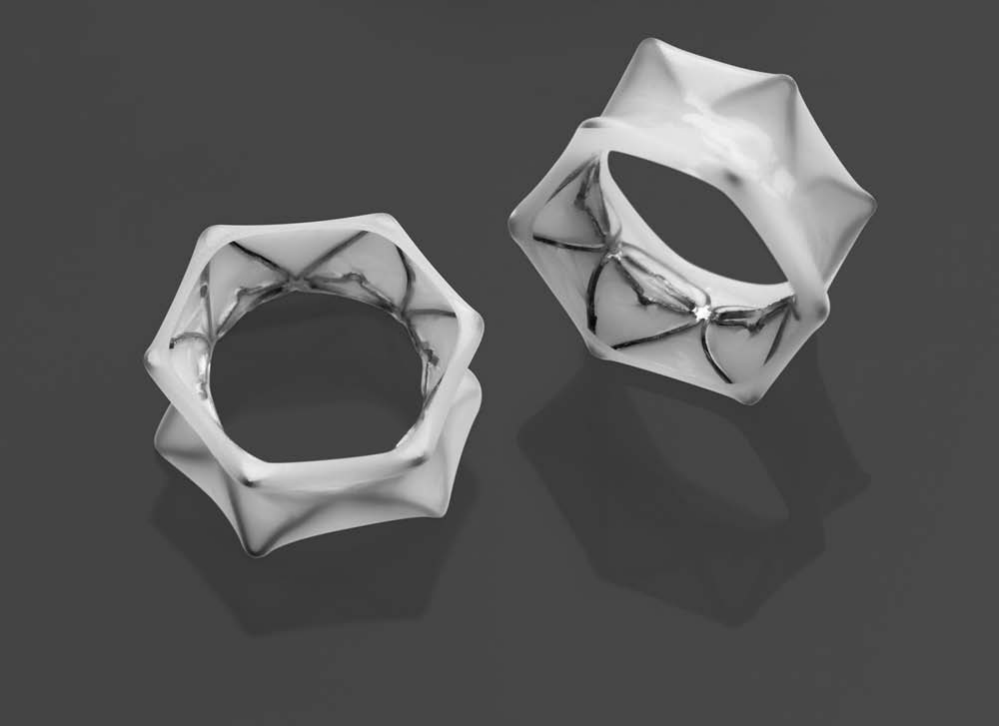
**Exhale Drug-Eluting Stents**.

An Exhale DES supports the newly-created passage connecting the lung tissue to the natural airway. The Exhale DES is pre-loaded on a balloon delivery catheter (Figure 3) which expands to place the stent (Figure 4) in the new passage. The Exhale Drug-Eluting Stent (3.3 mm inner diameter channel, 5.3 mm outer diameter with the stent deployed, with a flare at each end, 2 mm in length) is composed of stainless steel and silicone that contains the drug paclitaxel, which is intended to inhibit fibrotic or other tissue growth in the passage. The drug elutes into the airway wall and lung tissue over time, with most of the drug released during the first month following the procedure.

**Figure 5:**
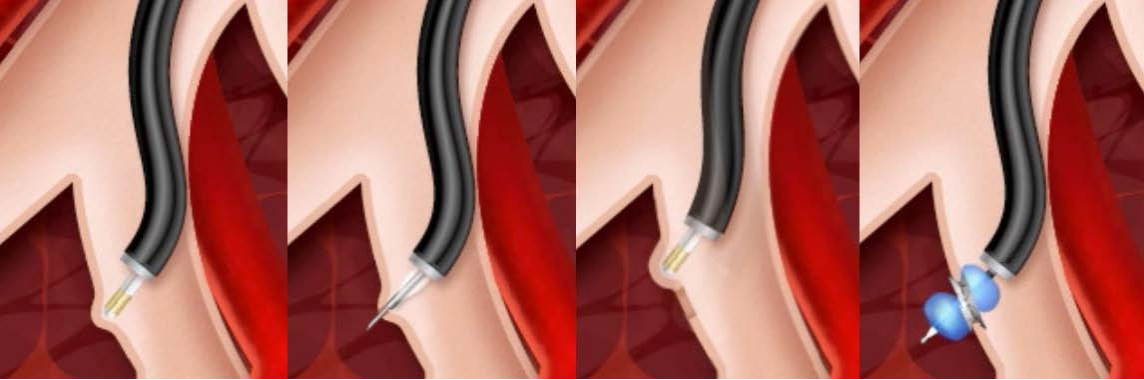
**Airway Bypass procedure steps**.

The Airway Bypass procedure (Figure 5) involves 4 different steps: 1) A Doppler probe scans the selected airway area in order to find the quiet spot in which there are no identifiable sounds of blood flowing and thus no major blood vessels; 2) The airway wall is then pierced with the transbronchial needle, the needle is retracted, the catheter is advanced and the dilating balloon is inflated to widen the passage; 3) The passage and adjacent area are rescanned with the Doppler probe to confirm the absence of surrounding blood vessels; 4) The needle is withdrawn and the catheter containing the drug-eluting stent is positioned within the passageway and the stent deployed by an inflating balloon.

### Inclusion/Exclusion Criteria

Table 1 summarizes the inclusion criteria which are similar to most of the criteria used in the National Emphysema Treatment Trial (NETT) (3).

**Table 1:** EASE Trial Inclusion Criteria

History and physical	Age ≥35 years
	Subject agrees to consult with a cardiologist prior to randomization if findings determine the need for such a consultation
HRCT scan	Patient diagnosed by radiology core lab with evidence of homogeneous emphysema with a total score of ≥8
Pulmonary Function	Post-bronchodilator ratio of FEV_1_/FVC <70%
	Post-bronchodilator FEV_1 _≤50% of predicted or FEV_1 _< 1 liter
	Post-bronchodilator RV >180% of predicted
	Post-bronchodilator RV/TLC ≥0.65 at screening
Dyspnea	Marked dyspnea, scoring ≥2 on the modified Medical Research Council scale of 0-4, confirmed at the end of pulmonary rehabilitation
Arterial blood gas analysis	PaO_2 _≥45 mmHg on room air
Rehabilitation	Supervised pulmonary rehabilitation of 16-20 sessions over 6-10 weeks prior to the scheduled study procedure
Smoking	A smoking history of at least 20 pack-years (1 pack year = 1 pack/day for 1 year) and subject has stopped smoking at least 8 weeks prior to signing the informed consent
Consent	Subject has provided written informed consent using a form that has been reviewed and approved by the Institutional Review Board or Ethics Committee
Follow-up	Subject is willing to be maintained on standard medical therapy for emphysema for 12 months following the procedure
	Subject is willing and able to return for all required follow-up and supervised pulmonary rehabilitation visits following study enrollment

Exclusion criteria (Table 2) are also similar to those used in NETT. The potential for electrical interference from the Exhale DPU during Airway Bypass poses a potential limitation for subjects with implanted defibrillators or pacemakers and therefore, these subjects cannot participate in the study.

**Table 2:** EASE Trial Exclusion Criteria

Pulmonary Function	Change in FEV_1 _> 20% between pre-and post-bronchodilator measurements or >200 mL if the subject's post-bronchodilator FEV_1 _< 1 liter
	Clinically significant bronchiectasis
	Three or more respiratory infections requiring hospitalization in the last 12 months
	Respiratory infection <30 days prior to randomization
	Presence of segmental atelectasis, lobar consolidation, significant or unstable pulmonary infiltrate or pneumothorax confirmed on x-ray
	DLco <15% of predicted
Arterial blood gas analysis	Values on room air, pH <7.35 with a PaCO_2 _> 50 mmHg or PaCO_2 _> 60 mmHg regardless of pH
Physical	BMI >31.1 for males and >32.3 for females
	Unplanned weight loss >10% usual weight in 90 days prior to randomization
General Medical	Uncontrolled hypertension (systolic >200 mmHg or diastolic >110 mmHg)
	Stroke within last 12 months
	Suspicion or history of pulmonary hypertension, defined by either of the following: Abnormal Radionuclide Ventriculogram/Echocardiogram showing Right Ventricular Ejection Fraction <30%; or evidence of right ventricular dilatation; or evidence of hypokinesia; or RVSP >45 mmHg
	Myocardial infarction within 6 months
	Type 1 diabetes
	Current diagnosis of renal failure
	Lung cancer or pulmonary nodule requiring surgery
	Ventilator dependence
	Previous lung volume reduction surgery or lobectomy
	Known hypersensitivity to aspirin, paclitaxel or stainless steel

Pulmonary function tests listed in the inclusion/exclusion criteria are performed according to standards that have been jointly developed by the American Thoracic Society/European Respiratory Society (ATS/ERS), and the National Emphysema Treatment Trial Manual of Operations.

### Radiology assessment

Both thick and thin slice CT scans were obtained at full inspiration (total lung capacity) and at the end of expiration (residual volume). The scanners were evaluated for each of the centres by performing a CT scan on a phantom. The approval process and radiological assessment was performed by the core lab (MedQIA, Los Angeles, CA, USA) using a novel standardized computer assisted diagnosis technique with quantitative image analysis). The radiology core lab quantified the percent of emphysema in a lobe using the objective Density Mask method in which the percent of emphysema is defined as the percentage of voxels with attenuation values below a specified level in a given lobe. Attenuation values of ≤-910 Hounsfield units (HU) from a thick section (10 mm) scan are considered emphysematous. The severity and distribution of emphysema are determined from high-resolution computer tomography (CT) scans in a manner similar to the definition used in NETT: Each lobe of the lung, except the middle lobe, is assessed and assigned a grade of 0, 1, 2, 3 or 4 based on the percent of lung destruction within the lobe (as shown in Table 3). Homogeneous emphysema is defined as a difference in scores of less than two between the two lobes *in at least one lung*, with an overall score of ≥8. (Note that using the NETT criteria, if one lung was scored to have heterogeneous emphysema, then that subject was categorized as heterogeneous.)

**Table 3:** Emphysema Lobar Grading

Lung Grade	Percentage of Lung Destruction within the Lobe
0	No lung destruction
1	1-25% lung destruction
2	26-50% lung destruction
3	51-75% lung destruction
4	76-100% lung destruction

### Medical treatment and pulmonary rehabilitation

Throughout the duration of the trial, the standard medical care management program all subjects receive is consistent with the recommendations of the American Thoracic Society/European Respiratory Society ATS/ERS Standards for the Diagnosis and Management of Patients with COPD (updated 2005). Prior to baseline testing, the pulmonary rehabilitation program consists of completion of 16-20 supervised exercise sessions over 6 to 10 weeks, ideally attending 2-3 pulmonary rehabilitation sessions per week. Completion of maintenance pulmonary rehabilitation or 16-20 sessions is an inclusion criterion for the trial. Post-operatively, 10 supervised exercise sessions over 8-9 weeks is required.

### Bronchoscopy

The blinding of investigators requires two trial teams: One performing the Airway Bypass and sham bronchoscopy procedures (Bronchoscopy Team), whereas a separate blinded team (Assessment Team) conducts the post-procedure follow-up evaluations on all subjects. To preserve the blinding, review and discussion of all post-procedure chest x-rays and CTs are conducted in the absence of Assessment Team members. Bronchoscopy is performed under general anesthesia or deep sedation with amnesic properties. Investigators use the same sedation or anesthetic protocols for treated and control subjects alike. The control group subjects are sedated and undergo bronchoscopy as if they were receiving Airway Bypass, but no passages are created or stents placed. Bronchial washings for culture are obtained first, followed by Doppler scanning in the right middle lobe. The bronchoscope is then retracted to a point below the vocal cords and above the carina. Simulated stent insertion is performed in this region and the duration of the sham procedure is at least one hour.

In the treatment group, up to 6 Exhale DES (optimally a minimum of 1 per treated lobe, maximum 2 per treated lobe, 6 overall) are placed. The right middle lobe and any lobe that has a CT score of zero are not treated. The number and location of the placed stents is determined by the investigator's visual assessment of the anatomic features of the airways and the amount and type of tissue destruction. Stent placements are targeted to segmental airways leading to regions where tissue destruction and air trapping are noted in the inspiratory and expiratory CT scans. In addition to this subjective assessment, for each case (treatment or sham) the bronchoscopist is provided with a radiologist report that includes lobular volume data and an analysis of specific segmental airways in the lung lobes (3 segments in the right upper lobe, left upper lobe and left lower lobe and 5 segments in the right lower lobe) from the core lab. For each segment the analysis includes measurements in millimeters of the proximity to tissue destruction, airway diameter, and average airway wall thickness. The presence of blood vessels is also noted. It is expected that regardless of plan for stent placement, the presence of blood vessels or an inability to place a stent could result in a stent located in a nearby but perhaps less desirable airway.

### Post-procedure

Subjects are expected to remain in the hospital and monitored for at least 1 night following their intervention. Prior to being discharged, and at follow-up visits at months 1, 3, 6, and 12, all subjects are asked to complete a "Study Subject Questionnaire" to assess the success of the blind before any testing is performed at each visit. Following the procedure, study subjects undergo another 8-9 weeks of supervised pulmonary rehabilitation.

Subjects in both control and treatment arms undergo evaluations at months 1, 3, 6, and 12 (Table 4). After all tests are completed (at the 12-month visit), subjects are told if they are in the treatment or the control group. Treated subjects are asked to return once a year for 4 more years for examinations and testing. The study is subject to intent-to-treat analysis and all reasonable efforts are made to contact terminated subjects for follow-up data collection for the first 12 months.

**Table 4:** Survey, Examination Schedule and Clinical Parameters

Procedure	Initial Screening	Baseline Testing	Procedure Day	Post-Procedure Days 1-2	Month 1 ± 7 days	Month 3 ± 14 days	Month 6 ± 14 days	Month 12 ± 14 days	Years 2-5 ± 60 days (treated subjects)
Health Inventory		X		X	X	X	X	X	X

HRCT Scan performed in certified scanner	X^1^			X^5^			X	X^7^	

Chest X-Ray	X^2^		X^5^						

Pulmonary Rehabilitation - Supervised	16-20 sessions over 6-10 weeks			After discharge for 8-9 weeks					

Cotinine, carboxyhemoglobin, or carbon monoxide	X^2^	X^3^				X			

Photograph	X^2^			X			X		

Blinding assessment questionnaire				X	X	X	X	X	

St. George's Respiratory Questionnaire		X^3^			X	X	X	X	X

Quality of Well-being scale		X^3^			X	X	X	X	X

Medical History (or interval history)	X^2^				X	X	X	X	X

ASAPSC (Anesthesia score)		X							

Dyspnea Index (modified mMRC)	X^2^	X^3^		X	X	X	X	X	X

Physical Exam, to include RR, HR, SpO_2_		X^3^			X	X	X	X	X

Electrocardiogram		X^3^							

Spirometry	X^2^	X^3^		X	X	X	X	X	X

Body Plethysmography	X^2^	X^3^		X	X	X	X	X	X

DLco	X^2^	X^3^			X	X	X	X	

Echocardiography to rule out pulmonary hypertension and congestive heart failure	X^2^								

Pregnancy test (women of child-bearing age)		X^4^							

CBC, Blood Chemistry Panel		X^3^			X	X	X	X	

PTT/INR/PT (only if on acticoagulation therapy)		X^3^							

Serum Creatinine		X^3^							

Arterial Blood Gases	X^2^	X^3^					X		

6-Minute Walk (with BORG scores)		X^3^			X	X	X	X	

Cycle Ergometry (work output)		X^3^					X		

Intraveneous antibiotic			X						

Begin aspirin			X (post op)						

Begin oral antibiotic and give subject pocket ID card				X					

^1 ^Within 6 months prior to randomization				^5 ^As soon as possible ***following ***treatment, and interpreted by the Bronchoscopy investigator (***not ***the Assessment investigator)					
						
^2 ^Within 4 months prior to randomization									

^3 ^Within 4 weeks prior to randomization and following at least 6 weeks of supervised pulmonary rehabilitation				^6 ^Required pre- and post-op.					

^4 ^Within 24-hours **prior to **intervention day				^7 ^CT scan at 12 months for Airway Bypass subjects only					

An independent Data Safety and Monitoring Board evaluates the progress of the study and assesses all adverse events. The safety stopping rules are described in Table 5.

**Table 5:** Safety Stopping Rules

Adverse Event	Stopping Rule
Major hemoptysis	> 3 of first 100 subjects then
	> 3% subjects or
	> 2 subjects/study site
Respiratory failure	> 15 of first 100 subjects then
	> 15% subjects or
	> 2 subjects/study site
Pneumothorax	> 4 of first 20 subjects then
	> 20% subjects or
	> 2 subjects/study site
Death	≥2 of first 66 subjects within 30 days post procedure or >3% thereafter

The primary safety endpoint is a comparison of a composite of five severe adverse events (SAEs) endpoint between the treatment and control groups. Composite safety is computed for each subject at each follow-up visit. The composite score recognizes a subject as having a safety concern if one or more of the five SAEs in Table 6 have been reported during the scheduled duration of the study.

**Table 6:** Safety Outcomes

1	Major hemoptysis: ≥200 mL estimated blood loss or requiring transfusion, or requiring arterial embolization, or surgical/endoscopic intervention
2	Respiratory failure requiring mechanical ventilation ≥24 hours
3	Pulmonary infection or COPD exacerbation requiring hospitalization >7 days
4	Pneumothorax requiring tube drainage >7 days
5	Death within 30 days of device implantation or the initial hospitalization if longer than 30 days, and death from respiratory causes

### Statistical Analysis

A Bayesian adaptive approach to the sample size selection is used with various operating characteristics and simulations performed to evaluate statistical power. A minimum total sample size of 225 and a maximum of 450 are considered. An interim data look is made when 225 subjects have been accrued. If trial success is highly likely accrual will be stopped. If accrual continues, another look is made after 45 additional subjects have been accrued. These 45 subject increment looks continue until accrual is stopped or 450 subjects are accrued.

To determine effectiveness, the treatment arm (Airway Bypass) is compared to the control arm (sham bronchoscopy). The two primary efficacy outcomes, FVC and mMRC, are combined in a responder analysis. A subject is a success (responder) if their FVC improves by at least 12% of their baseline value and their mMRC improves (is reduced) by at least 1 point at their 6-month follow-up visit. In order for superior efficacy to be claimed, the probability of a subject being a responder in the treatment arm must be greater than the control arm. The primary efficacy analysis will be on an intent-to-treat basis on all subjects who enter the procedure room for intervention.

Secondary endpoints will be analyzed using 6 month data. A responder analysis will be done separately for mMRC and for FVC. Only those subjects with complete 6-month data will be included in this analysis. Sensitivity analyses will be done to investigate the possible effects of missing data. The secondary endpoint Residual Volume/Total Lung Capacity (RV/TLC) will be analyzed for superiority. Other secondary endpoints analyzed are: RV, FVC, mMRC, FEV_1_, St. George's Respiratory Questionnaire, 6-minute walk test, and cycle ergometry.

**Figure 6:**
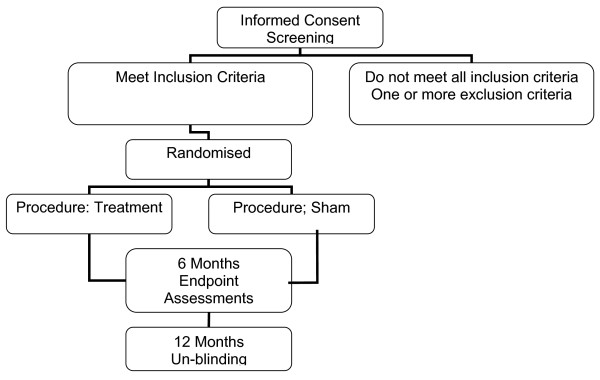
**CONSORT flowchart**.

For subjects who are excluded between randomization and procedure the following information will be collected and reported to the US Food and Drug Administration: reason for dropout, randomization assignment, and baseline data. The patients and A team investigators are un-blinded at 12 months (Figure 6).

## Discussion

A preliminary clinical evaluation of the Exhale DES in a previous open-label protocol has demonstrated 6 month improvement in pulmonary function and subjective improvement in some subjects (4). This largest clinical study of Airway Bypass published that thirty-five emphysema subjects had an average of 8 Exhale DES implanted. Six months after the procedure, the mean decrease for all subjects was a statistically significant 400 mL in RV from baseline (p = 0.04) and 0.5 points (p = 0.025) in mMRC dyspnea score. The median RV/TLC ratio for the subjects at baseline was 0.67. For the most hyperinflated subjects (defined as those above this median) the decrease in RV at 6 months was 870 mL (p = 0.022) and mMRC also decreased by 0.5 points (p = 0.035). While the 17.8% improvement in FVC over baseline for this group was not statistically significant, it was higher than the 12% generally recognized as clinically significant (5).

A variety of factors may influence the study outcome. Correct technical placement of stents is important and in itself is influenced by a number of factors such as bronchoscopic accessibility and vascularity of the target sites. The duration of benefit of a successfully-placed stent also remains unclear. Finally, the effect of the 2 to 1 randomization (treatment to sham) may also influence the results. Where randomization to treatment is greater than a 1 to 1 ratio there may be a greater placebo effect, as most patients believe they have been randomized to treatment. This is the first randomized trial of endoscopic treatment designed specifically for patients with homogenous emphysema. Since patterns of response in such patients have not been yet studied, we anticipate that this trial will bring to light new aspects and add to the current knowledge yielding to further investigation of endoscopic procedures for emphysema.

## Competing interests

Broncus Technologies funded the EASE trial and the authors received research support. PLS: The Royal Brompton Hospital was reimbursed for all clinical trial expenses by Broncus;

DJS: University Medical Center Groningen was reimbursed for all clinical trial expenses by Broncus; PFGC: Santa Casa de Porto Alegre-Pavilhao Pereira Filho Hospital was reimbursed for all clinical trial expenses by Broncus; EC: The Royal Brompton Hospital was reimbursed for all clinical trial expenses by Broncus; GWS, Meizinische Universitatsklinik, Saarland was reimbursed for all clinical trial expenses by Broncus; JDC: is a consultant for Broncus and the Hospital of the University of Pennsylvania was reimbursed for all clinical trial expenses by Broncus.

## Authors' contributions

JDC participated in the design of the study. PLS and DJS developed the initial draft of the manuscript. All authors contributed to subsequent revisions. The contributing authors have read and approved the final manuscript.

## Pre-publication history

The pre-publication history for this paper can be accessed here:

http://www.biomedcentral.com/1471-2466/11/1/prepub
